# A systematic review of medication non-adherence in persons with dementia or cognitive impairment

**DOI:** 10.1371/journal.pone.0170651

**Published:** 2017-02-06

**Authors:** Daisy Smith, Janaka Lovell, Carolina Weller, Briohny Kennedy, Margaret Winbolt, Carmel Young, Joseph Ibrahim

**Affiliations:** 1 Department of Forensic Medicine, Monash University, Southbank, Victoria, Australia; 2 Ballarat Health Services, Ballarat, Victoria, Australia; 3 Department of Epidemiology and Preventive Medicine, Monash University, Victoria, Australia; 4 Australian Centre for Evidence Based Aged Care, College of Science, Health and Engineering, La Trobe University, Victoria, Australia; Banner Alzheimer's Institute, UNITED STATES

## Abstract

**Background:**

Adherence to medication is vital for disease management while simultaneously reducing healthcare expenditure. Older persons with cognitive impairment (CI) are at risk for non-adherence as cognitive processes are needed to manage medications. This systematic review focuses on the relationship between medication non-adherence and specific cognitive domains in persons with CI, and explores determinants of medication non-adherence. When available, relationships and factors are compared with cognitively intact populations.

**Methods:**

A seven database systematic search of studies published between 1 January 1949–31 December 2015 examining medication non-adherence in community dwelling persons with CI or dementia was conducted. Articles reporting medication non-adherence in people with CI or dementia in the community, with or without caregiver supports were eligible for inclusion. Papers reporting adherence to treatments in cognitively intact populations, populations from hospital or institutional settings, for non-prescribed medication or those describing dementia as a factor predicting medication non-adherence were excluded. Data on study and population characteristics, research design, data sources and analysis, specific cognitive domains, non-adherence prevalence, measurement of adherence, salient findings, factors associated with adherence and strategies to improve medication adherence were extracted. Study limitations included inconsistencies between data sources and definitions, resulting in a loss of fidelity in the value and comprehensiveness of data, as well as exclusion of non-pharmacological treatments and regimens.

**Findings:**

Fifteen studies met inclusion criteria. Adherence among CI subjects ranged from 10.7%-38% with better rates of adherence in non-CI individuals. Medication non-adherence definitions varied considerably. New-learning, memory and executive functioning were associated with improved adherence and formed the focus of most studies. Multiple factors were identified as modulators of non-adherence.

**Conclusion:**

This review highlights a gap in knowledge on how specific cognitive domains contribute to medication non-adherence amongst CI populations, and demonstrates the current focus is limited to two domains: memory and executive functioning.

## Introduction

In 2008, chronic diseases accounted for 63% of global deaths [1]. As of 2013, there are approximately 117 million individuals in the USA with one or more chronic diseases, placing a significant burden on health care costs [2]. Self-management provides the patient with more control and responsibility to achieve effective disease management while simultaneously reducing healthcare expenditure [3].

Effective management of chronic comorbid conditions often involves complex medication regimens, requiring different tablet combinations and multiple daily dosing [4]. There is a high rate of non-adherence to medication regimens, particularly in patients with chronic conditions [5]. Fortunately, adherence may be improved through a combination of patient educational and behavioural interventions [6]. Older people are at risk of non-adherence due to a normal decline in dexterity, mobility, hearing and vision; however, impaired cognitive function may exacerbate these effects [7, 8]. Of note, there is a paucity of research literature investigating the impact of dementia on the ability of patient’s adhering to complex medication regimens [9].

Deficits in cognitive processes due to dementia predisposes older adults to medication non-adherence by impairing abilities in planning, organising and executing medication management tasks [10, 11]. Multiple patient, environmental and systemic factors may also modulate medication adherence [8]. To date, no single factor has accounted for more than a modest explanation for non-adherence [12]. Disease features, referral process, clinical settings, therapeutic regimen, patient demographics, treatment factors (cost, dosing frequency, side effects etc.) inconsistently explain non-adherence and thus cannot be used to adequately predict it [13].

The impact of dementia on a patient’s ability to self-manage varies according to the cognitive domain(s) affected (Table 1), severity of impairment, and complexity of the self-management task(s). The functions of multiple cognitive domains are required to adhere to medication regimens [14, 15] as this task involves obtaining and accessing medications, understanding directions, scheduling intake, adjusting schedules, planning continuous access to medication and problem-solving missed doses[16, 17]. Deficits in any cognitive domain(s) will impact a persons’ ability to adhere to prescribed medication subsequently resulting in medication errors, medication related hospital admissions, and dependence on others to assist with medication management tasks [11, 18]. Furthermore, feasible long term interventions to improve medication adherence in chronic disease is lacking [19].

**Table 1:** DSM V Criteria for Diagnosing Major & Minor Neurocognitive Disorder (NCD)*.

Cognitive Domain †	Description
Complex attention	Includes sustained attention, divided attention, selective attention and information processing speed.
Executive function	Includes planning, decision making, and working memory, responding to feedback, inhibition and mental flexibility.
Learning and memory	Includes free recall, cued recall, recognition memory, semantic and autobiographical long term memory, and implicit learning.
Language	Includes object naming, word finding, fluency, grammar and syntax, and receptive language.
Perceptual-motor function	Includes visual perception, visuoconstructional reasoning and perceptual-motor coordination
Social cognition	Includes recognition of emotions, theory of mind and insight.

*Dementia newly defined as Major NCD; CI newly defined as Minor NCD in DSM-V.

†Cognitive domains retrieved from *https://fightdementia.org.au/files/helpsheets/Helpsheet-DementiaQandA11-DiagnosticCriteriaForDementia_english.pdf*.

Research on specific cognitive domains and medication management tasks are predominantly focused on learning, memory and executive functioning, with little research into the remaining domains (attention, language; perceptual-motor function and social cognition) [20, 21] (Table 1). A comprehensive understanding of the influence of all cognitive domains on non-adherence is necessary for clinicians to improve care.

## Aims

The aim of this systematic review is to elucidate the relationship between medication non-adherence and specific cognitive domains in persons with dementia/CI. The secondary aim is to determine factors related to medication non-adherence in persons with dementia/CI who take medication for treatment of comorbid chronic disease(s).

## Methods

### Definitions

This review has adopted the clinical diagnostic criteria of the Diagnostic Statistical Manual version 5 (DSM-V) to define dementia and cognitive impairment and outline the specific cognitive domains (Table 1) [22].

### Study selection

Eligibility criteria encompassed articles reporting medication non-adherence in people with CI or dementia in the community, with or without caregiver support.

Inclusion criteria comprised original research in peer-reviewed journals available in English language between 1 January 1949–31 December 2015. Studies with participants who had dementia as described by authors and comorbid chronic diseases were included. Articles reporting dementia of different severities were also included. Article definitions and methods of diagnosing dementia were not restricted to the DSM-V clinical diagnostic criteria used to structure this review.

Excluded were studies on medication non-adherence in hospital or institutional setting (e.g. nursing home). Papers reporting adherence to treatments other than prescribed medication and those that described dementia as a factor predicting medication non-adherence were excluded. We also excluded study populations consisting of cognitively intact persons only.

### Reporting guidelines

The systematic review was conducted in accordance with the Preferred Reporting Items for Systematic Reviews and Meta-Analyses (PRISMA) Statement (PRISMA-P checklist)[23] (S1 Table).

Terms used in this review describing cognitive impairment, dementia and medication adherence are outlined in Table 1.

### Data sources and searches

The following seven databases were selected: Ovid MEDLINE, EMBASE, CINAHL (via EBSCOHOST), COCHRANE DATABASE OF SYSTEMATIC REVIEWS, PsycINFO (via EBSCOHOST), Web of Science, and Scopus.

The search conducted on 14^th^ October 2015, used explosions and combinations of key search terms (S2 Table).

Search results were collated in a reference data base (EndnoteX5, Thomson Reuters, 2010), duplicates deleted and initial screening of titles was independently conducted by two reviewers (DS & CY). A priori inclusion and exclusion criteria were applied at this stage. Two reviewers (DS & BK) then independently screened abstracts of titles retained by at least one reviewer, to select final studies to include. Two reviewers independently applied inclusion and exclusion criteria to full texts of remaining references to select studies for this review (DS & JI). Manual searches of reference lists and citation tracking of papers identified as potentially relevant were also conducted. Discordance between reviewers was resolved by discussion and when necessary, by a third senior reviewer (JI).

### Data extraction and quality assessment

Extracted data included study and population characteristics, research design, data sources and analysis, specific cognitive domains, prevalence of non-adherence, method of measuring adherence, salient findings, factors associated with adherence, strategies for improving adherence and study limitations. When available in the research literature, relationships and factors are compared with cognitively intact populations. Coding decisions were made by agreement between two researchers (JI & DS). Information on demographics, prevalence of adherence, methods for promoting adherence and potential risk factors for non-adherence were collated. Internal validity of included articles was assessed using the National Institutes of Health (NIH) study quality assessment tool comprising 14 criteria. Two reviewers independently rated each study against the criteria before an overall quality rating was assigned to each study (Table 2, S1 Appendix, S3 Table) [24].

**Table 2:** Methods and Populations of Selected Studies.

			Methodology				Setting and Population		
Author, year	Aim	Country	Design	Data source	Setting	Study period (years)	Population	No. of persons with CI/Dementia (%)	Quality of studies
Foebel, 2012	Role of caregivers and caregiver stress in medication adherence in older home care clients with MCI	CAN	R, Co	HC, Sur, MR, St, Fam/CG,	State/ County	2006–2007	Persons with heart failure, MCI & caregivers	59,662(42%)	G
Mackin, 2006	Determine the relative contribution of measures of cognitive functioning and mood status on treatment adherence	USA	R, Co	HD, HC, Sur, Int	State/ County	-	Older adults at primary care clinics	29%	F
Poon, 2009	Evaluate the utilization of and adherence to antihypertensive and dementia medications in a cohort of veterans across different racial/ethnic groups	USA	R, Co	HD, MR, Sur	National	2000–2005	Veteran with a diagnosis of both hypertension and dementia	56,561 (100%)	G
Hawkins,2012	Describe the cognitive domains affected in patients with CI, examine clinical and demographic variables potentially associated with CI, and to determine the relationship between CI and MA	USA	P, Co	HC, MR, Int, St	State/ County	2009–2011	English speaking veterans. No subjects had known CI before study enrolment (N = 251)	144(58%)	F
Thiruchselvam,2012	Examine the influence of cognitive, medical, behavioural, and social risk factors on medication NAD in community-dwelling older adults with CI	CAN	P, Co	Sur, MR, HC, Int, Fam/CG	National	1997–2005	Older adults with CI whom lived alone and took at least one medication	339 (100%)	F
Smith, 2007	Assess telehealth home monitoring system.	USA	P,Co	Sur, Fam/CG, Int,MRD	National	1998(6mnts)	People with mild dementia who live alone and took ≥1 medications daily. Three groups: video, phone, control.	14 (100%)	F
Kamimura, 2012	Test efficacy of medication reminder device in medication management for elderly patients with MCI	USA	P, Co	Fam/CG, MRD, Int	National	2008–2011	Elderly with MCI	18(100%)	G
Conn, 1994	Assess patients taking drugs for co-morbid disease to determine whether this had a role in slowing further cognitive decline.	USA	P, Co	MR,Int	State/ County	-	Persons with CI (≤23 MMSE score) and non-impaired controls (N = 178)	35 (20%)	F
Insel, 2006	Examine the relationship between adherence and measures of executive function or working memory and memory.	USA	P,Co	Sur	National	-	Community-based older adults taking daily prescribed medications.	95	G
Boucher, 1996	Describe problems of dementia patients with CI spousal caregivers.	USA	Cc	HC, Int, Sur, Fam/CG	National	1992–1994	Dementia patients	AD: 56 (86%); Other: 9 (14%)	G
Cotrell, 2006	Examine the relationship between patients’ cognitive status, deficit awareness, medication management skills, and actual medication adherence.	USA	Cc	Int, Sur, Fam/CG	State/ County	-	Persons with AD and healthy controls. Caregivers were also included (N = 47)	27 (57%)	G
Fulmer, 1997	Examine the potential usefulness of the Medication Management Test (MMT)	USA	Cs	Sur, Int, Fam/CG	State/ County	-	CI elders and cognitively normal elders whom had a reported caregiver (N = 125)	51 (41%)	G
Okuno,2001	Examine whether CI is a risk factor for non-adherence	JAP	Cs	Sur, Int	State/ County	1998–2000	Community dwelling functionally independent elderly living	58(26%)	G
Cameron,2010	Test the impact of CI on self-care.	AUS	Cs	Int, MR, Sur	State/ County	20007–2008	Persons with Chronic HF	68 (73%)	F
Stoehr,2008	Explore associations between two specific cognitive domains and aspects of medication management	USA	Co	Sur, Int, HC, MR	State/ County	1999–2001	Older primary care patients. MMSE scores ≤25 and a control group of ≥25	343	G

**General:** (-) Not stated/specified.

**Country:** USA = United States of America;; CAN = Canada; AUS = Australia; JAP = Japan.

**Design:** R = Retrospective; P = Prospective; Cs = Cross-sectional; Ob = Observational; Co = Cohort; RCT = Randomised controlled trial, Cc = Case Control.

**Data source:** Sur = Survey, Int = Interview, St = Staff, Fam/CG = Family/Caregiver, HC = Healthcare personnel, MR = Medical records, HD = Health database, MRD = Medication reminder device.

**Dementia type:** MCI = Mild cognitive impairment; AD = Alzheimer’s Disease; CI = Cognitive impairment/ed.

**Adherence:** NAD = non-adherence/; ADH = adherence/t.

## Results

### Study selection

**Fig 1:**
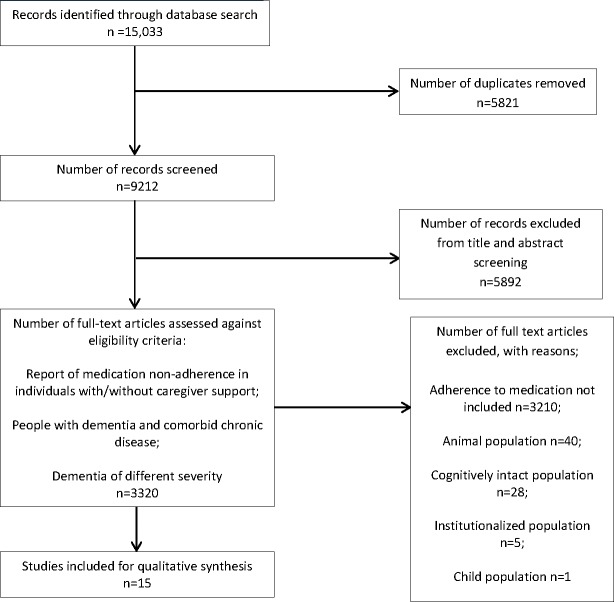
PRISMA Flow Diagram Identification, Screening, Eligibility and Included Articles.

The combined searches yielded 15,033 records of which 15 articles were eligible for inclusion (Fig 1).

### Study characteristics

The first included study was published in 1996, and the majority (n = 11) were conducted in the USA [14, 18, 21, 25–32]. Studies were cohort (retrospective, n = 3), cohort (prospective, n = 7), cross sectional (n = 3), and case control (n = 2) in design (Table 2). Data collection methods included interviews (n = 12), databases (n = 2), surveys (n = 1), through interactive video technology (n = 1) and electronic recording devices (n = 1). The quality of studies was rated as good (n = 7), fair (n = 7) and poor (n = 1). (Table 2) [24].

Adherence in persons with dementia living in the community was the focus in eleven studies [20, 25–29, 31–35], and of these, three examined the role of caregivers of CI older adults [25, 28, 34]. The remaining studies (n = 4) described adherence in the general population with a subgroup of cognitively impaired persons [14, 18, 21, 30]. Studies were too varied in purpose, design and sample to be analysed in an aggregate form. Two studies also collected qualitative data [29, 32] using semi-structured interviews to obtain information about medication management devices from family/caregivers.

Sample sizes ranged from 8 [32] to 56,561 [31] participants. Participant demographics were relatively homogenous; age in years of late 70s to early 80s, predominately female [14, 20, 21, 25, 26, 29, 34, 35], Caucasian [14, 18, 26, 30, 31], completed 11–12 years of education [14, 20, 21, 25, 30, 32], had a diagnosis of dementia or mild cognitive impairment (MCI) [18, 25–27, 29, 31–35] and lived with a spouse or family member [18, 25, 26, 28, 34]. There was a wide range in the level of cognitive impairment in participants between the studies, with 9.6 [34] to 100% [25] of participants with severe CI and 29 [30] to 72.2% [29] with MCI. Methods and population of selected studies are further outlined in Table 2.

### Medication non-adherence and adherence

The definition of medication non-adherence and adherence varied widely (Table 3) with studies describing under and overtaking (n = 2), omission of a single dose (n = 7), deviation from a prescribed time (n = 2) and deviation from dose intervals (n = 3).

**Table 3:** Findings of Selected Studies.

			Cognition		Adherence			
Author, year	CI associated with NAD	Method of ascertaining CI	Severity of CI	Domain(s) affected	Definition of non-adherence	Method of determining non-adherence	Adherence rates (n)	Other risk factors for non-adherence
Foebel, 2012	Y	CPS	M: 43.3% S: 9.6%	-	<100% ADH with medication indicated on assessment item & missed appointments	Medication use in past 7 days	Distressed caregivers and independent living were 2.95 times more likely to be NAD than those with non-distressed, at-home caregivers.	-
Mackin, 2006	Y	DRS	M: 29%	A, VC, LM,	-	Physician rating &self-report	Specific cognitive domain contribution recorded not ADH rates	Poor performance on memory subscale of DRS
Hawkins, 2012	Y	SLUMS	M: 104 (41.6%) S: 40 (16%)	A, I, VC, LM, L	ADH score (%) w/o cut-off score for NAD	Pill count	Those with MCI 70% ADH, severe CI 73%. ADH.	Unrecognised CI
Boucher, 1996	Y	BRDS (Patient) &KOMCT (Caregiver)	S: 65 (100%)	-	Pill count comparison	Pill count	42% ADH for patients with demented caregivers	Demented caregivers
Fulmer, 1997	Y	CMSQ	M / S : 51 (41%)	-	-	MMT; Caregiver report	35 (70%) of patients with CI reliant (advice, assistance, giving) with regards to administration of medication	Poor MMT scores
Insel, 2006	Y	MMSE & Additional cognitive tests and subtests	NGM/R	LM, E	% of days correct no. of doses taken	Electronic medication monitoring cap	62% ADH to medication at least 85% of the time.	Poor executive working memory score; Poor MMSE
Stoehr, 2008	Y	Neuropsychological test battery	NGM/R	LM, E	<50% prescribed doses OR omission of any 1 medication	Self-reports; Direct inspection; Semi structured interview	Among those taking ≥1 drugs, 71% took all their medications regularly as prescribed.	Higher no. of prescription drugs; Higher dosing frequency; Lower scores on tests of working memory
Cotrell, 2006	Y	MMSE (for AD patients without current score)	S: 27 (57%)	-	Deviation from predicated ADH	Pill count	ADH in the AD group ranged from 17% to 100%.	CI; No assistance; verbal/visual assistance
Poon, 2009	Y	Medical Records Review	S: 56,561 (100%)	-	MPR < 0.8	Pill counts; Interview	ADH in all drug classes lower in African Americans compared with Caucasians. Being Hispanic was associated with lower ADH rates for some drug types compared with Caucasians.	African American: lower ADH all classes exc. ARBs, K+ sparing diuretics & Loop directs. Hispanic: lower ADH for CCBs and AchEinhibtors
Thiruchselvam, 2012	Y	DRS: Dementia & CI score < 130	M / S: 339 (100%)	A, AB, VC, LM, E	≥1 incident of over/under dosing of medication	Independent rater review	17.4% had at least one incident of medication NAD reported	Previous occurrence of NAD; ≥4 medications; Increase in certain DRS subset scores
Okuno, 2001	Y	MMSE: CI score <24	S: 58 (26.4%)	-	ADH rate <80%	Pill count	Poor ADH rates (<80%): 76 (34.6%)	CI; medication concern; educational; Initially self-selected prescribed drugs; no medication calendar; poor relationship with physician
Cameron,2010	Y	MMSE: CI score <26–27 &MoCA: score <26	M / S: 68 (73%)	-	-	Interview (6 +/- 5 days after hospitalization)	Inadequate self-care maintenance: 43 (47%)	Experience with CHF < 2 months; MCI; Comorbidity index
Conn, 1994	N	MMSE: CI score <23	M / S: 35 (20%)	-	Pills usually missed per week	Pill count and self-report	Relation between CI and ADH/NAD recorded not MA	-
Kamimura, 2012	-	MMSE & CDR	MMSE—M: 13 (72.2%); S: 5 (27.8%) CDR–M:10 (55.6%); S: 8 (44.4%)	-	Elderly with MCI	SAMR prior to device use & at 1 and 3 months after use	Ability to use medication reminder device not MA recorded	-
Smith, 2007	-	MMSE: MCI score 24–27; Dementia score <24	M / S: 14 (100%)	-	-	Pill count	ADH rates in the video-monitored group remained stable whereas phone group and control group declined	Phone intervention and no telehealth home monitoring

**General:** (-) Not stated/specified.

**CI associated with non-adherence:** Y = Yes; N = No.

**Severity of CI:** M = Mild Cognitive Impairment; S = Severe Cognitive Impairment; NGM/R = No global Measurement/Reporting of Cognitive Impairment.

**Methods of ascertaining CI:** CPS = Cognitive Performance Scale; DRS = Dementia Rating Scale; MMSE = Mini-Mental State Examination; SLUMS = Saint Louis University Mental Status (SLUMS) examination; BRDS = Blessed-Roth (functional) Dementia Scale; KOMCT = Katzman Orientation-Memory-Concentration Test; CMSQ = Comprehensive Mental Status Questionnaire.

**Domains affected:** A = Attention; AB = Abstract Reasoning; VC = Visual and Constructional; LM = Learning and Memory; E = Executive Function; I = Information Processing; L–Language.

**Definition of non-adherence:** MPR = Medication Possession Ratio.

**Method of determining non-adherence:** MMT = Medication Management Test; SAMR = Self-administration Medication Rate.

**Dementia type:** MCI = Mild cognitive impairment; AD = Alzheimer's Disease; CI = Cognitive impairment.

**Adherence:** NAD = non-adherence; ADH = adherence.

Six studies described the frequency of medication non-adherence [14, 18, 20, 21, 27, 28]. The frequencies of non-adherence varied considerably across studies. The smallest rate was 10.7% among CI older adults aged 65 or more years using surveys in a cohort study [21]. The greatest rate was 38% of participants *“falling below the adherence threshold of taking medication correctly 85% of the time”* in one prospective cohort study using electronic monitoring [14]. Adherence frequencies using ‘pill counts’ ranged from 17%-100% among older adults with Alzheimer’s Disease (AD) from one case control study[27].

#### Under and overtaking medications

Two prospective cohort studies reported under and overtaking medications were common [18, 20]. In one study, 17.4% of participants reported at least one incident of medication non-adherence during the 12-month prospective follow up period [20]. These comprised one incident (14.7%), two incidents (2.4%) and three incidents (0.3%) [20]. The other study, examined all domains (excluding abstract reasoning) in participants with heart failure who screened positive for CI [18]. Through pill counts of all prescribed medication, this found that MCI and severe CI persons were 30% and 27% non-adherent (pills were not taken, over taken or a combination of both) respectively within a 30 day period[18].

#### Omission of a single dose

Omission of a single dose was specified in seven studies [14, 18, 21, 25, 26, 32, 34]. When reported, adherence frequencies fell to 42%-71%. The majority used pill counts to determine medication adherence (n = 4) whilst the remaining used self-reports (n = 1), survey (n = 1) and a medication-monitoring device (n = 1). Most studies did not specify cognitive domains and instead used a global description of CI (n = 4) [25, 26, 32, 34].

Three studies specified cognitive domains including, attentional [18], speed of information processing [18], visuospatial and constructive skills [18], new learning and memory [14, 18, 21], receptive and expressive language [18], praxis [18] and executive functioning [14, 18, 21] (Table 3).

#### Deviation from prescribed time/dose intervals

Deviation from a prescribed time and deviation from dose intervals was documented in one case control study using a global description of CI [27]. The control (non-CI) group performed better in tasks of timing and dosing compared to participants with AD. Participants with AD also over-estimated their ability to time and dose medications correctly (92% self-predicted versus 78% actual, respectively) whereas controls were accurate at both tasks.

### Overview of the relationship of non-adherence with specific cognitive domains/deficits

Five articles investigated deficits in cognitive domains: attention (n = 3) [18, 20, 30], speed of information processing (n = 1) [18], visuospatial and constructional skills (n = 3) [18, 20, 30], praxis (n = 1) [18], new learning and memory (n = 5) [14, 18, 20, 21, 30], executive functioning (n = 5) [14, 18, 20, 21, 30] abstract reasoning [20] as well as receptive and expressive language [18] (Table 3).

Methods and instruments for assessing the relationship between adherence and cognitive domains varied. Two studies utilized the Dementia Rating Scale (DRS) [20, 30] while two studies used the Mini Mental State Examination (MMSE) in conjunction with additional cognitive tests and subtests [14, 21].

#### Attentional and speed of information processing

Attention [18, 20, 30] and speed of information processing [18] were not statistically significant predictors of medication adherence and management. These were prospective [18, 20] and retrospective cohort studies [30] using the DRS [20, 30] and the Saint Louis University Mental Status (SLUMS) examination to ascertain cognitive impairment [18].

#### Visuospatial and constructional skills and praxis

Visuospatial [18, 20, 30] or praxis [18] test scores were not statistically associated with medication adherence however, one study found participants often knew medications by colour and shape rather than name or indication [18].

#### New learning and memory and executive functioning in medication adherence

Most studies focused on memory (n = 5) and executive function (n = 5) [14, 18, 20, 21, 30] when assessing medication adherence.

Three studies reported better memory as a significant predictor of adherence [18, 20, 30] while two studies did not [14, 21]. CI participants were used in two of the studies reporting significance [18, 20]. The remaining studies did not focus on CI populations. Two studies used the same methods to ascertain memory scores [20, 30] finding better performance on the DRS memory subscale a predictor of better medication adherence [20] and poor performance a predictor of missed medical appointments [30]. Furthermore, CI was the sole factor associated with poor medication adherence.

CI participants who completed further neuropsychological testing in one study demonstrated deficits in immediate memory and delayed verbal memory [18]. Intact executive functioning was protective for adherence in two studies [14, 21] and was determined through different forms of testing: Wisconsin Card Sorting Test (WCST); WMS III letter- number sequence, mental control and digit span backward [14] and Trailmaking B Test [21]. These recruited participants showing signs of CI (e.g. MMSE scores <25) [21] or deficits in domains indicative of impairment [14]. The remaining studies, using CI population samples, reported poorer executive performance increased the likelihood of non-adherence (n = 1) [20] or was not statistically significant (n = 2) [18, 30]. Interestingly, two used the DRS initiation/preservation subscale [20, 30].

#### Abstract reasoning

One prospective cohort study reported better performance on the DRS conceptualization subscale increased the likelihood of non-adherence [20].

#### Receptive and expressive language

There was not a significant association between language domain tested and medication adherence in one prospective cohort study [18].

### Factors associated with non-adherence

#### Individual factors

Eleven studies documented individual factors associated with medication adherence and non-adherence in cognitively intact and cognitively impaired individuals [14, 18, 20, 21, 26, 27, 30, 31, 33–35] (Table 4).

**Table 4:** Significant Factors for Medication Adherence and Non-Adherence in Cognitively Intact and Cognitively Impaired Individuals.

Cognition									
	Cognitively Intact			Cognitively Impaired			Common to Cognitively Intact & Cognitively Impaired		
	Factor	Direction of Association		Factor	Direction of Association		Factor	Direction of Association	
**Adherence**	MMT Score	⇧	r = 0.44; p < 0.00 ^[^^24^^]^	Global Impairment	⇩	OR 2.94; 95% CI: 1.32–6.58 ^[^^35^^]^; ß = -0.25; r = -0.27; p < 0.01 ^[^^33^^]^	Intentional	⇩	OR 19.65; 95% CI 9.22–41.92 ^[^^35^^]^
				MCI	⇩	Adherence: 70.7% vs no Ci:78.1% vs dementia: 73.3%; 95% CI: 63–78.4; df (5.68 (1)); p = 0.017 ^[^^18^^]^	Poor Physician Relationship	⇩	OR 3.55; 95% CI 1.55–25.20 ^[^^35^^]^
				≥ 5 prescription drugs	⇩	OR 0.45; 95% CI: 0.21–0.95 ^[^^21^^]^	Forgetting	⇩	r_s_ = -0.40; p = 0.0001 ^[^^26^^]^
				Ethnicity (AF & H vs. W)	⇩	p < 0.05 ^[^^31^^]^	↓ DRS M	⇩	ß = -0.381; t = -2.681; p = 0.010 ^[^^30^^]^
				Ci Spousal CG	⇩	ADH: 42% vs. Non-Ci spousal CG ADH: 83% p = 0.041 ^[^^25^^]^	↑ GDS Total Score	⇩	ß = 0.436; t = 2.608; p = 0.012 ^[^^30^^]^
					No Assistance	⇩	- ^[^^27^^]^	Co-morbidity Index	⇧	ß = 0.21; r = 0.23; p < 0.01 ^[^^33^^]^
				Verbal/Visual Assistance	⇩	- ^[^^27^^]^	Disease Severity	⇧	ß = 0.19; r = 0.20; p < 0.01 ^[^^33^^]^
				EF (TTB Score)	⇧	OR 4.38; 95% CI: 1.13–9.33 ^[^^21^^]^	Exp. with disease (HF) > 2mo	⇧	ß = 0.31; r = 0.31; p < 0.01 ^[^^33^^]^
				MMT Score	⇧	r = 0.39; p < 0.03 ^[^^28^^]^	EF & WM	⇧	ß = 0.44; t = 3.05; p < 0.05 ^[^^14^^]^
				Televideo Monitoring	⇧	80–81% vs. NM: 62% p < 0.05 ^[^^32^^]^			
				Min Assistance with Medication	⇧	- ^[^^27^^]^			
				Physical Assistance	⇧	- ^[^^27^^]^			
				Medication reminder device	⇧	- ^[^^29^^]^			
**Non Adherence**				Previous Non-Adherence	⇧	OR 2.61; 95% CI: 1.18–5.62 ^[^^20^^]^			
				≥4 Medications	⇧	OR 2.58; 95% CI: 1.31–5.29 ^[^^20^^]^			
				Caregiver Stress & NLWC	⇧	OR 2.95 ^[^^34^^]^			
	Not assessed by included studies			↑ DRS C Score	⇧	OR 1.14; 95% CI: 1.02–1.27 ^[^^20^^]^	Not assessed by included studies		
				↑ DRS IP Score	⇩	OR 0.93; 95% CI: 0.87–1.00 ^[^^20^^]^			
				↑ DRS M Score	⇩	OR 0.89; 95% CI: 0.81–0.97 ^[^^20^^]^			
				Co-morbidity (HF)	⇩	- ^[^^34^^]^			
				Age	⇩	- ^[^^34^^]^			

**General:** p = p-value; OR = Odds Ratio; Exp. = Experience; Med = Medication; ADH = Adherence; ß = Beta Coefficient; r = Correlation Score; CI = Confidence Interval

**Factors:** Ci = Cognitive Impairment; MCI = Mild Cognitive Impairment; DRS = Dementia Rating Scale; NLWC = Not living with client; GDS–Geriatric Depression Scale; Ed = Education; NM = No Monitoring; CG = Caregiver; Reln = Relationship; TTB = Trailmaking Test B Score; MRD = Medication Reminder Device; MMT = Medication Management Test

**Direction of Association:** ⇧ = Factor increased adherence/non-adherence in this population; ⇩ = Factor decreased adherence/non-adherence in this population; HF = Heart Failure; AF = African American; H = Hispanic; W = White

**Domain Tested:** C = Conceptualization; IP = Initiation/Perseveration subscales; M = Memory; WM = Working Memory; EF = Executive Functioning

Individual hazardous factors reported: African American and Hispanic ethnicity [31]; forgetting [21, 26]; lower scores in cognitive domain test indicative of deficits [14, 20, 21, 30]; dementia/CI [18, 27, 33, 35]; depression [30]; inadequate self-care confidence [33]; lower level of education, concern about taking prescribed drugs and intentional noncompliance [35].

Of note, global dementia/CI scores using MMSE [14, 26] were not associated with adherence in two prospective cohort studies and when reported, where total MMSE score ranged from 14–23 (M = 20.72) [26]. Presence of dementia was associated with not knowing the name and purpose of medications, having others assisting and preparing medications and an adult child assisting with medications [26].

#### Dyad/Carer

The likelihood of participants with CI having a caregiver to assist with medications was reported in six studies [25–29, 34]. Presence of caregiver established self-administration dependence and low capacity of self-medication [26, 28] as well as improved adherence [27, 34]. Informants were able to accurately predict the care recipient’s adherence rates and performance on medication management tasks in one case-control study [27].

Participants with CI were more likely to have someone prepare medication [26, 27, 29]. Hazardous factors affecting medication adherence for CI persons included caregiver distress, not living with a caregiver [34], absence of assistance (e.g. reminders, pill box check and setting-up lists) [27] and spouses as primary caregivers who were also cognitively impaired [25].

#### Medication

Three studies reported medication factors in relation to non-adherence [20, 21, 35]. Taking fewer drugs was associated with improved adherence in one study using self-reports [21] and CI persons taking four or more daily medications had a 2.5 fold increase in non-adherence compared to those taking less than four medications according to independent rater-reviews [20]. Conversely, a cross-sectional study reported a non significant association between medication adherence and: the number of drugs; or frequency of drug administration; or with/without one dose package; or use of medication calendar or written drug information using pill counts [35].

#### Medication aids

A memory assistive device was used more often by CI participants (32/35) than non-CI participants (132/143) [26]. The lack of such device was associated with non-adherence [35]. Better scores on memory subscales were also associated with participants setting up their own medication schedules [21]. Additionally, environmental cues associated with the repetitive task of taking medication were reported to have contributed to increased adherence [14].

#### Healthcare system factors

Health system factors were reported in three studies [21, 30, 35]. There was a negative correlation between physician rating and patient’s ratings of medication treatment adherence [30]. Furthermore, poor client-physician relationships was an independent predictor of poor adherence (defined as <80%) [35] whilst prescription insurance was positively associated with medication adherence on univariate analysis only [21].

### Interventions or strategies used to manage medications

Technological intervention specific to medication non-adherence were described in two studies [29, 32]. Another two studies identified participant’s strategies to manage medications [21, 26].

Telecommunication technology [32] and a medication reminder device [29] were used to assess medication self-administration in participants with dementia. These suggested that participants could become proficient users of such interventions [29, 32]. Video monitoring intervention stabilized adherence even as global mental status declined over time, while adherence for the control group (no monitoring) declined as global mental status declined [32]. End-of-study adherence was statistically significant for the video monitored group (81%) compared to controls (62%) [32].

One study reported 89% (n = 231/257) participants with an assistive system to track medication were fully adherent. This proportion was similar to those not using assistive methods 87% (n = 74/86) [21]. The most common assistive system was specific placement of medications to trigger memory (34.2%, n = 92). These findings were discordant with another study, whereby CI participants’ MMSE scores were not associated with reported use of memory focused assistive methods [26]

## Discussion

### Statement of key findings

Poor adherence to medication regimens in people with CI ranged from 10.7% [21] to 38% [14] while adherence levels ranged from 17% to 100% among older adults with Alzheimer’s Dementia [27].

Frequencies of adherence in these CI populations were worse when compared to cognitively intact populations [14, 21].

Interestingly, when an informal caregiver was ensuring adherence, the objective adherence rates were similar in cognitively intact and impaired populations [27].

Aggregate analysis and direct comparison was limited because of inconsistencies and variations in definitions of adherence and non-adherence. Also the methods of ascertaining adherence were disparate [36].

To our knowledge, this is one of very few systematic reviews to deconstruct cognitive functioning and identify specific domains associated with medication adherence [21].

### Interpretation

#### Cognitive domains

Specific cognitive domains receiving the most attention were memory and executive functioning. Studies with CI populations found intact memory was a significant predictor of medication adherence [18, 20]. Any associations with executive functioning remain unclear due to discordant study results. Persons with CI may not be able to understand, retain or follow instructions, implying that interventions focusing on traditional models of patient education may fall short in this population [18].

The impacts of a better level of executive performance in persons with CI on medication adherence were discordant, one reported improvement [20] and another did not [18].

In contrast, studies with populations considered cognitively intact reported better executive function to be a significant factor for adherence [14, 21]. These discordant results may be due to differing methods utilized to test this domain. Executive functions correlate with instrumental activities of daily living requiring goal directed activities [37] suggesting executive abilities involving mental flexibility, including implementing, planning and maintaining intentions, may be important for medication adherence [20].

Interestingly, better performance on the subscale of abstract reasoning increased the likelihood of medication non-adherence in a sample of CI older adults. This may be explained by intentional non-adherence due to individuals’ concerns about the medication [35] as this requires the ability to abstract and form fundamental connections between medication and possible side effects–the ability measured by the selected subscale [20]. There was not a significant association between language domain tested and medication adherence [18]. However, subtle impairments in verbal memory amongst cognitively intact women taking oral anti-estrogen therapy was a potential predictor of non-adherence [38]. Such findings have implications for clinical practice. For example, interventions to improve adherence could potentially benefit from providing written instructions and resources[38], not just verbal and the use of assistive technologies. Furthermore, it has implications for the use of brief screening tools to more efficiently identify at-risk patients for closer monitoring and the development of assessment tools to inform targeted adherence interventions[38].

These findings need replicating, though physicians should be aware of the error in assuming impairment in all areas of cognitive functioning will increase non-adherence. Health care providers need to explore CI patient’s understanding and concerns regarding medications. This may reduce a patient’s intentional non-adherence. Education about indication, medication and side effects, along with considering patient tolerance of side effects may improve intentional non-adherence [20]. Other specific cognitive domains received much less attention, with inconsistent or non-significant findings of those examined [18, 20, 30].

It is surprising that impairment in specific domains was not as useful in understanding and preventing medication non-adherence in this population [18, 20, 30]. Further research is needed to understand this complex relationship [15, 39] and elucidate if different patterns of suboptimal adherence may exist depending on the combinations of neurocognitive impairment [39].

#### Risk factors

Most frequently reported risk factors for medication non-adherence were CI (or suspected CI) and absence of a caregiver or spouse living with the patient. The impact of impairment on adherence begun with MCI and did not worsen for participants with severe CI. This is important for clinical practice as the presence of MCI is easily missed [40]. Although it is not feasible to screen all older peoples for CI [41], clinicians should be aware of the relationship of these risk factors to non-adherence. Methods of ascertaining dementia or CI varied amongst the studies making it difficult to compare results. The assistance of a caregiver may potentially indicate advanced dementia/CI and may provide an explanation why a relationship between cognitive functioning and medication adherence is not reported [26]. Research should further explore these risk factors in order to reliably ascertain adherence.

Studies that did not find a relationship between dementia or CI and adherence most frequently used self-reports or interviews to ascertain adherence, both tending to lead to under-estimation of medication non-adherence [9]. These findings may be biased because of inherent methodological limitations as patient self-reports and interviews of CI persons are poor measures of treatment adherence [42]. Future studies should consider the use of other methods to identify adherence/non-adherence in an older population, including personalized medical records and direct pharmacist questioning, which have been suggested as optimal measurement tools in previous studies [43].

A significant number of people with dementia have a comorbid health condition, which may have serious implications for the way specialist services are delivered to people with dementia [44]. Regimen complexity [21]and the number of prescribed medications [20] were cited as risk factors for medication non-adherence. Physicians should be aware of the relationship between medication adherence and comorbidity, a factor likely to increase and complicate medication intake. Patients with multiple chronic disease may vary their opinions about health outcomes, such as, longer survival, prevention of disease-specific events, physical and cognitive function and tolerable risk of adverse drug reaction[45]. The difficulty for clinical practise is to rationally prescribe medications for older adults with multiple chronic conditions and reduced life expectancy whilst also analysing: the likelihood of benefit and goals of care and satisfying the basic principles of optimal medication use. Therefore, future research should focus on a subpopulation of persons with dementia or CI with co-morbid diseases [46].

#### Interventions

Unfortunately, interventions were investigated by only two studies [29, 32] which had small sample sizes.

A recurring theme is the importance of caregivers for the success of interventions [29]. This, however, reinforces the dependency of older people with dementia and is inconsistent with the philosophy of promoting self-determination and independence critical to a person’s quality of life [47]. Individual strategies (e.g. medication regimens or setting up schedules) used in two studies [21, 26], led to recruitment of willing participants with caregivers, hence may not represent the general older population.

Several interventions (such as medication and disease education, medication reviews and packaging/dispensing of medications) for the general older adult population have been documented. Despite moderate success, few studies have attempted to translate these findings to older adults with dementia or CI. This study updates and extends the knowledge of a previously published systematic review [9]. This study included a more comprehensive search to retrieve a broader scope of articles and focuses on the impact of specific cognitive domains on medication adherence.

Research into the effect of a memory-prompting device designed for participants with HIV-associated memory impairment demonstrated improvements in adherence to highly active-antiretroviral therapy (HAART) for these participants but not for memory-intact participants [48]. These results suggest different approaches are necessary for each population.

#### Medical consequences

Medication non-adherence, particularly overdose, may result in toxicity due to altered pharmacodynamics in the older population [49]. Well-known consequences: poor disease control, increased hospitalizations, disability and early death [20] were rarely examined in the appraised studies. Research in this field tends to exclude persons with dementia/CI, limiting the ability to extrapolate results to the growing cognitively impaired population [8, 9].

### Strengths and limitations

This is an extensive review with a comprehensive search strategy and was not limited to quantitative research. Studies were scored using recognized reporting standards, determining that only one [26] of the 15 reviewed met less than half of the specified criteria. Limitations were the inconsistencies between data sources and definitions, resulting in a loss of fidelity in the value and comprehensiveness of data gathered by each method. Finally, non-pharmacological treatments and regimens were excluded. Articles that were written or translated into English were only able to be included in this review.

### Implications

Clinical practice must take into account the accumulating research for the prevention of medication non-adherence and the management strategies available for this population. Medication reminder devices are suggested to combat this issue, however, the degree of efficacy of these devices and the appropriate support for using such a device amongst this population are yet to be determined [29].

Given the paucity of data available, future research could explore a realist review approach to combine theoretical understanding and empirical evidence. A realist review focuses on explaining contextual relationships between how interventions are applied and produce outcomes [50]. This may enable a deeper understanding of potential effectiveness of interventions while waiting for empirical clinical study evidence.

This study identified several methodological gaps and highlights the lack of focus on specific cognitive domains that may potentially contribute to medication non-adherence. There is also a paucity of information about adherence and dementia subtypes.

### Generalizability

Generalizing the findings should be done with caution. The eligible research studies spans over 20 years (1994–2012) and the nature and assessment of medication adherence/non-adherence have changed along with changes in diagnosis of dementia and CI as well as health care practice.

Individual-level factors are possibly transferable as demographic characteristics are similar across the CI population. As most studies were conducted in the USA, there may be issues with the applicability of clinical practice or research factors being translated to over countries.

## Conclusion

This systematic review consolidates current knowledge about medication non-adherence in persons’ with dementia/CI. The literature revealed poor cognitive function as a risk factor of medication non-adherence. It also highlighted the importance of caregivers in assisting with medication adherence or interventions to improve medication adherence. Clinicians should be aware of the negative effect global cognitive impairment has on medication adherence and consider screening patients where impairment is indicated. Development of tailored interventions to combat non-adherence requires a better understanding of the potential contribution of cognitive domains. This is also requires researchers to develop and use a single standard definition and method of recording medication adherence. The development of knowledge about medication non-adherence in persons with dementia/CI is vital if the challenge of ensuring better prognoses and reduced harm to patients is to be met.

## Supporting information

S1 Appendix NIH Quality Assessment Tool Criteria and Ratings. (DOCX)

S1 Table PRISMA checklist. (DOCX)

S2 Table Search terms used in literature search. (DOCX)

S3 Table Quality Ratings per paper appraised in current study. (DOCX)
