# Supplementary material for: A systematic review of medication non-adherence in persons with dementia or cognitive impairment
Source: PLoS One. 2017 Feb 6;12(2):e0170651. doi: 10.1371/journal.pone.0170651 (PMC5293218; doi:10.1371/journal.pone.0170651)
Supplement: S1 Appendix — (DOCX) [file pone.0170651.s001.docx]

**S1 Appendix NIH Quality Assessment Tool Criteria and Ratings.**

1. Was the research question or objective in this paper clearly stated?
2. Was the study population clearly specified and defined?
3. Was the participation rate of eligible persons at least 50%?
4. Were all the subjects selected or recruited from the same or similar populations (including the same time period)? Were inclusion and exclusion criteria for being in the study specified and applied uniformly to all participants?
5. Was the sample justification, power description or variance and effect estimates provided?
6. For the analyses in this paper were the exposure(s) of interest measured prior to the outcome(s) being measured?
7. Was the timeframe sufficient so that one could reasonably expect to see an associated between exposure and outcome if existed?
8. For exposures that can vary in amount or level did the study examine the different levels of the exposure as related to the outcome (e.g. categories of exposure measured as continuous variable)?
9. Were the exposures (independent variables) clearly defined, valid, and reliable and implemented consistently across all study participants?
10. Was the exposure(s) assessed more than once over time?
11. Were the outcome measures (dependent variables) clearly defined, valid, and reliable and implemented consistently across all study participants?
12. Were the outcome assessors blinded to the exposure status of the participant?
13. Was the loss to follow up after baseline 20% or less?
14. Were key potential confounding variables measured and adjusted statistically for their impact on the relationship between exposure(s) and outcome(s)
