# Supplementary material for: A systematic review of medication non-adherence in persons with dementia or cognitive impairment
Source: PLoS One. 2017 Feb 6;12(2):e0170651. doi: 10.1371/journal.pone.0170651 (PMC5293218; doi:10.1371/journal.pone.0170651)
Supplement: S2 Table — (DOCX) [file pone.0170651.s003.docx]

**S2 Table Search terms used in literature search.**

| **Adherence (n=11)** | **Medication (n=10)** | **Dementia (n= 6)** | **Medication adherence (n=9)** |
| --- | --- | --- | --- |
| Adherence | Medication | Dementia | Medication adherence |
| Persistence | Prescription drug | Alzheimer's Disease | Medication error |
| Compliance | Drug therapy | Cognitive impairment | Treatment compliance |
| Nonadherence | Treatment | Memory disorders | Medication compliance |
| Non-adherence | Prescription | Mild cognitive impairment | Medication management |
| Patient compliance | Medicines | Impaired cognition | Drug discontinuation |
| Patient adherence | Treatment regimen |  | Adherence treatment |
| Patient nonadherence | Treatment schedule |  | Adherence schedule |
| Patient non-adherence | Medication safety |  | (non-adher* adj2 (drug* or medicat* or prescription* or pharmaceutical*)).tw. |
| Concordance | Pharmaceutical |  |  |
| Non-concordance |  |  |  |

The search was checked and finalised by a librarian with substantial experience in databases search
