# Supplementary material for: A systematic review of medication non-adherence in persons with dementia or cognitive impairment
Source: PLoS One. 2017 Feb 6;12(2):e0170651. doi: 10.1371/journal.pone.0170651 (PMC5293218; doi:10.1371/journal.pone.0170651)
Supplement: S3 Table — (DOCX) [file pone.0170651.s004.docx]

**S3 Table Quality Ratings per paper appraised in current study.**

Legend: Y = Yes; N = NO; CD = Can’t determine; NA = Not applicable; NR = Not reported
